# Assessing the limitations of relief-based algorithms in detecting higher-order interactions

**DOI:** 10.1186/s13040-024-00390-0

**Published:** 2024-10-01

**Authors:** Philip J. Freda, Suyu Ye, Robert Zhang, Jason H. Moore, Ryan J. Urbanowicz

**Affiliations:** 1 Computational Biomedicine, Cedars-Sinai Medical Center, 700 N. San Vicente Blvd., Pacific Design Center, Suite G540, West Hollywood, 90069 CA USA; 2 Whiting School of Engineering, Johns Hopkins University, 3400 N. Charles St., Baltimore, 21218 MD USA; 3 University of Pennsylvania, Philadelphia, 19104 PA USA

**Keywords:** Epistasis, Feature selection, Heterogeneity, High-order, Interactions, RBA, Relief-based algorithm, ReliefF, Univariate

## Abstract

**Background:**

Epistasis, the interaction between genetic loci where the effect of one locus is influenced by one or more other loci, plays a crucial role in the genetic architecture of complex traits. However, as the number of loci considered increases, the investigation of epistasis becomes exponentially more complex, making the selection of key features vital for effective downstream analyses. Relief-Based Algorithms (RBAs) are often employed for this purpose due to their reputation as “interaction-sensitive” algorithms and uniquely non-exhaustive approach. However, the limitations of RBAs in detecting interactions, particularly those involving multiple loci, have not been thoroughly defined. This study seeks to address this gap by evaluating the efficiency of RBAs in detecting higher-order epistatic interactions. Motivated by previous findings that suggest some RBAs may rank predictive features involved in higher-order epistasis negatively, we explore the potential of absolute value ranking of RBA feature weights as an alternative approach for capturing complex interactions. In this study, we assess the performance of ReliefF, MultiSURF, and MultiSURFstar on simulated genetic datasets that model various patterns of genotype-phenotype associations, including 2-way to 5-way genetic interactions, and compare their performance to two control methods: a random shuffle and mutual information.

**Results:**

Our findings indicate that while RBAs effectively identify lower-order (2 to 3-way) interactions, their capability to detect higher-order interactions is significantly limited, primarily by large feature count but also by signal noise. Specifically, we observe that RBAs are successful in detecting fully penetrant 4-way XOR interactions using an absolute value ranking approach, but this is restricted to datasets with only 20 total features.

**Conclusions:**

These results highlight the inherent limitations of current RBAs and underscore the need for the development of Relief-based approaches with enhanced detection capabilities for the investigation of epistasis, particularly in datasets with large feature counts and complex higher-order interactions.

**Supplementary Information:**

The online version contains supplementary material available at 10.1186/s13040-024-00390-0.

## Background

Feature selection, the process of reducing the dimensionality of a dataset while preserving or enhancing predictive power, is a critical step in machine learning (ML) [1], particularly when applied to computational genetics and bioinformatics. Genetic datasets often contain hundreds of thousands to millions of variants, representing single nucleotide polymorphisms (SNPs) or structural variants. These variants are encoded ordinally, with each containing an allele count, usually for the minor allele, scored as 0, 1, or 2 for each individual [2, 3]. The primary goal in quantitative genetic studies is to identify which variants are significantly associated with a trait or disease state (phenotype) [2, 3]. For consistency with ML terminology, we refer to genetic variants as features and phenotypes as outcomes. Traditionally, single-feature approaches, including quantitative trait locus (QTL) analyses and genome-wide association studies (GWAS), have been widely applied to determine which features have strong univariate effects on outcomes. However, these methods are limited in that they do not inherently capture non-additive genetic variation, which includes interactions between features [2–4]. Non-additive variation has been observed to have significant contributions to variance explained in various systems, including humans [5–9]. More recently, ML approaches have been leveraged not only to identify associations that may be missed by traditional methods but also to handle the complexity and scale of genetic data [10–13]. ML algorithms can model non-linear relationships and interactions more effectively than traditional methods, making them well-suited for uncovering subtle and complex patterns in data [14]. Feature selection plays a crucial role in this process, as it enables the identification of the most relevant features while reducing dimensionality, thereby enhancing the performance and interpretability of the models [1].

In the context of genetic data, interactions between features, commonly referred to as epistasis, play a crucial role in understanding complex traits. Epistasis occurs when the effect of one feature is modified by one or more other features, leading to interactions that can either mask or enhance the combined effect [2, 3]. Epistasis can be understood in two distinct ways: statistical epistasis, which refers to the detection of interactions through significant results in linear or logistic models or via feature importance scores in ML algorithms, and biological epistasis, which describes the actual mechanistic interplay between features via biological pathways and functions [15]. Traditional approaches and ML techniques can detect statistical epistastic interactions which serve as a basis for follow-up validation studies aimed at explaining the underlying biological epistasis.

Despite its significance in the analysis of complex traits, epistasis is challenging to detect due to the exponential growth in possible interactions as the number of loci (*n*) considered in *k*-wise combinations increases. Simultaneously, the total combinations of *k* loci increases polynomially with *n* [16]. This expansion poses a formidable hurdle for feature selection, particularly when balancing the detection of univariate effects with the vast pool of potential interactions. Thus, developing a robust and efficient feature selection strategy is crucial for navigating and prioritizing features when employing ML techniques in the analysis of epistasis in large, complex genetic datasets.

There are various methods and software packages built to detect epistatic interactions in genetic data, each with their own strengths and limitations. A common shortcoming is that these approaches are exhaustive, requiring the generation of features or model terms for each *n* choose *k* interaction considered, making them either computationally inefficient or impractical for large-scale analyses. However exhaustive search (when practical) comes with the benefit of indicating “which” features are involved in a given interaction. Another drawback is that most methods focus on interactions, requiring separate tests to identify univariate effects. Linear mixed models (LMMs) are one method used for detecting epistasis [17], but exhaustive searches with LMMs are computationally expensive and prone to overfitting [17]. While programs like PLINK [18] and BitEpi [19], implemented in C++, are highly efficient, their computational demands increase significantly with the number of features and interaction orders. Additionally, PLINK’s epistasis function is limited to second-order interactions and assumes a multiplicative (Cartesian product) model for all interactions [18]. Different interaction models, such as exclusive-or (XOR), have been shown to yield varying epistatic results when compared to Cartesian [16]. Moreover, LMMs and PLINK incur significant multiple testing burdens as the number of interactions considered increases, severely restricting the detection of significant interactions. BitEpi, although not limited by statistical testing, can only be applied to case/control studies, not supporting multiclass or continuous outcomes [19]. Methods like BitEpi and Multifactor Dimensionality Reduction (MDR) [10–12] do not assume a strict model structure when constructing interactions or features, making them more sensitive to various types of feature interactions, but still rely on exhaustive search. Lastly, HOGLmine [20], based on the significant pattern mining framework [21], leverages existing knowledge on protein-protein interactions to guide searches and extends genotype encodings beyond additive models to enhance interaction sensitivity. However, HOGLmine also faces significant computational challenges as more features and higher orders are considered. Additionally, it suffers from the same statistical burdens as LMMs and PLINK, is restricted to case/control studies like BitEpi, and requires all SNPs within a genomic region to share the same encoding (e.g., dominant or recessive). Despite their strengths, these methods have notable weaknesses, highlighting the need for a more comprehensive and efficient approach to detect both univariate and epistatic effects.

Relief-based algorithms (RBAs), a family of filter-based feature selection methods, are popular for genetic analyses as they can detect both univariate effects and interactions without exhaustively searching the entire parameter space [22]. Furthermore, RBAs do not create new variables for each possible *n*-way interaction and operate independently of ML algorithms [13, 22], increasing overall efficiency and allowing them to be applied to any analysis pipeline. Additionally, RBAs do not rely on a specific encoding, and they can be applied to binary, multiclass, and continuous outcomes. Unlike other methods, RBA implementations are easily accessible through a user-friendly Python package, **skrebate**, a scikit-learn compatible [23] collection of RBAs.

RBAs achieve their efficiency and thorough evaluation by iteratively updating feature weights (or proxy statistics) to measure a feature’s relevance to the predicting outcome value based on the concept of ‘near hits’ and ‘near misses’ in the training set. These hits and misses are categorized by comparing feature value differences between instance pairs. The nearest neighbors of a particular instance that are of the same class (or similar outcome value in continuous outcomes) are called the *nearest hits*, while the nearest neighbors that are of the opposite class are called the *nearest misses*. These identified neighboring instances are then used to update feature weights. After execution, the RBA outputs the weight (feature importance) for each feature, which ranges from -1 to 1, prioritizing maximally positive features [24]. As a result, RBAs rank features based upon their relative predictive power and, depending on the RBA, score features in terms of univariate effects, interactions, or both. While we focus here on the effectiveness of RBAs in genetic datasets, these algorithms are versatile, supporting various contexts and accommodating datasets with categorical or continuous features, missing data, noisy data, and binary, multi-class, or continuous outcomes. Consequently, RBAs are widely used in fields beyond genetics, including healthcare analytics [25], image processing [26], finance [27], cybersecurity [28], and environmental science [29]. For a comprehensive overview of the implementation and applications of RBAs across different domains, see Urbanowicz et al. 2018 [22].

Due to the capability of RBAs to explore and detect both univariate and interaction effects and their limitations, a previous study benchmarked various RBAs within these contexts using a variety of simulated genetic datasets with known predictive features [13]. That study compared multiple RBAs on their capacity to detect univariate, epistatic, and heterogeneous effects. Although the capability and efficacy of feature interaction detection are dependent on the specific RBA employed, RBA algorithms identified features involved in 2-way and 3-way interactions with success under a variety of simulation scenarios. However, all RBAs failed to assign larger scores to predictive features involved in simulated higher-order (4-way and 5-way) interactions (using datasets with 20 features). Interestingly, in these experiments, the known predictive features were consistently given highly negative scores (often with all predictive features ranked with the most negative scores). Thus, these features would be incorrectly disregarded by standard RBA feature ranking. In-line with this observation, we previously speculated that there may be an opportunity to capture higher order interaction effects with an alternative score ranking approach [13].

To better understand the potential limitations of RBAs in detecting higher order interactions, it is helpful to revisit the foundational principles laid out in the original development of the Relief algorithm. In the original research paper introducing the Relief algorithm, Kira and Rendell posit that “statistically, the relevance level of a predictive feature is expected to be larger than zero and that of an irrelevant one is expected to be zero (or negative)” [24]. A subsequent study, however, indicates that predictive features might receive more negative scores under certain conditions [30]. Specifically, RBAs like ReliefF encounter difficulties in distinguishing between predictive and non-predictive (random) features as the number of nearest neighbors increases, particularly within noisy datasets with complex associations [13]. Initially, RBAs tend to assign negative scores to random features due to minor asymmetries in their update mechanisms - where differences with nearest neighbors from the same class lead to negative updates, and those from different classes result in positive updates. As the number of neighbors increases, these updates begin to balance out, often resulting in zero estimates for random attributes. Additionally, the presence of noise in the dataset further complicates the interpretation of these updates, potentially leading to the erroneous assignment of negative scores to predictive features. This influence of noise underscores the sensitivity of RBAs and raises considerations for their use in environments with complex higher-order interactions. Such over-generalizations can mask the true discriminative power of predictive features, especially as the algorithm’s sensitivity to noise escalates in high-dimensional settings. Consequently, in scenarios involving complex interactions, there may be a marked increase in assigning negative scores to genuinely predictive features, revealing either a critical limitation of RBAs or a potential opportunity that warrants further exploration to better define the boundaries of their interaction-detection capabilities.

A few questions arise from these previous observations: (1) Are RBAs indirectly detecting higher-order interactions (i.e., > 3-way) by assigning highly negative scores to predictive features? (2) Will this negative scoring of predictive features in higher order interactions persist in datasets with a larger number of features? And (3), Can absolute-value ranking of RBA feature scores be employed to capture high order interactions without significantly reducing their performance to detect univariate or 2 to 3 way interactions?

Detecting higher-order interactions in biological systems is a complex and challenging problem [16, 31]. Beyond the finite 4 and 5-way interaction results published in [13], the limitations of RBAs in detecting higher-order interactions, particularly in genetics, have not been clearly defined. Users employ RBAs with the expectation of detecting interactions, given their reputation as “interaction-sensitive” algorithms, but it is not yet clear up to what order of interaction they may be capable of performing reliably, and in what contexts (e.g., number of features, number of instances). The purpose of this study is to determine whether absolute ranking improves the ability of RBAs to detect higher-order feature interactions while still identifying single-feature and multi-feature associations, or if RBAs are inherently limited in this regard.

## Methods

### Feature importance estimation algorithms

We chose to evaluate ReliefF [32], MultiSURF [13], and MultiSURFstar [33] (hereinafter referred to as MultiSURF*), RBAs for this study, due to their reliable performance over a large variety of problem domains, with higher-order feature interactions being their most notable limitation [13]. We used the skrebate v0.62 Python package [13] which includes all three algorithms, and have made all analysis scripts available in release v0.7.1 on GitHub [34]. For the ReliefF algorithm, 10 vs. 100 nearest neighbors (*NN*) were examined for the run parameter traditionally labeled as *k*. To avoid confusion with the binomial coefficient notation (*n* choose *k*) we will henceforth refer to this run parameter *k* as *NN* to avoid confusion. These two values of *NN* replicate the analyses of the benchmarking paper [13] where *NN* = 10 yielded more positive scores for predictive features in higher-order experiments while *NN* = 100 yielded more negative scores [13]. We expect each setting of *NN* to yield different strengths and weaknesses. Particularly, in higher-order interactions, we would expect that ReliefF with *NN* = 100 will outperform ReliefF with *NN* = 10 when using absolute value ranking, as non-predictive feature weights should trend toward zero at higher *NN* settings (dependant on overall data sample size) [30]. In all figures, standard rankings for these RBAs are denoted as *ReliefF-10NN*, *ReliefF-100NN*, *MultiSURF*, and MultiSURF* while absolute value rankings are denoted as *ReliefF-10NN_ABS*, *ReliefF-100NN_ABS*, *MultiSURF_ABS*, and MultiSURF*_ABS.

#### ReliefF

ReliefF is an improvement over the original Relief algorithm [24, 35] and is now one of the most well-known and most used RBAs to date. In addition to discrete and continuous outcomes, ReliefF can handle multi-class and incomplete datasets as well [36, 37]. Unlike Relief, which only uses the single nearest hit and nearest miss to update feature weights, ReliefF requires the run parameter, *NN*, denoting the number of nearest neighbors that will be used for feature scoring. In each training cycle, a target instance is chosen, and the *NN* nearest hits and *NN* nearest misses to that target are then used to iteratively update feature scores [22, 30].

#### MultiSURF*

Instead of using a specific number of *NN* for scoring, MultiSURF* identifies nearest neighbors as well as ‘farthest instances’ using a boundary threshold, $T_i$, based on the mean pairwise distance between the target instance and others. It also uses a “dead-band” zone, based on the standard deviation of pairwise distances between the target instance and all others, to exclude ambiguously ‘near’ or ‘far’ instances from affecting feature scores. It was previously demonstrated that the inclusion of inverse-scoring for ‘far’ instances improved power to detect pure pair-wise interactions in Relief-based feature ranking [33, 38]. While effective, ‘far’ scoring requires additional computational cost, and was also demonstrated to negate the ability of Relief-based algorithms to detect univariate effects in feature ranking and selection [13]. This makes MultiSURF* only suitable for detecting 2 or 3 way interactions.

#### MultiSURF

MultiSURF uses the same threshold ($T_i$) and dead-band introduced in MultiSURF* to identify nearest neighbors, however it omits ‘far’ scoring. This reduces computational complexity as well as regains the efficacy of the algorithm to detect univariate effects, but at the expense of a small degree in power loss to detect 2 or 3 way interactions [13]. This makes MultiSURF efficient for broader applications, particularly in large datasets where simplicity and processing speed are crucial. This approach provides robust analysis while minimizing computational burdens and removing the need for hyperparameter optimization [13, 22].

### Score ranking schemes

We assess the performance of each aforementioned RBA in identifying known predictive features in simulated genetic datasets by utilizing two ranking schemes: standard and absolute value. For standard ranking, we sort feature scores in descending order from the most positive to the most negative. This is the traditional strategy used inherently by RBAs. In absolute value ranking, we instead first convert all feature scores to their absolute value and then rank in descending order, such that highly positive and highly negative scores are prioritized. The aim is to have one algorithm and ranking scheme that can capture the highly positive scores of univariate effects and lower-order interaction effects as well as the hypothesized highly negative scores for higher-order interaction effects.

### Data simulation

Table 1 presents a detailed overview of 2,100 simulated datasets with binary outcomes for classification, building on those used in a previous Relief-algorithm benchmarking study [13]. The ‘configurations’ column details all variations across experiments per genotype-phenotype association pattern, including the number of predictive and total features, heritability (indicative of noise level), the number of instances, and model architecture complexity-categorized as easy (E) or hard (H) [39]. Each configuration produces 30 replicate datasets using random seeds. Datasets are generated using custom scripts as well as the GAMETES v2.2 software package [40], which simulates a range of genotype-phenotype relationships, including univariate, multivariate, and pure, strict interaction effects. “Pure” refers to an epistatic interaction with no univariate effects, “strict” refers to no lower-order effects (e.g., a 3-way interaction has no lower-order 2-way interactions). These pure/strict models of epistasis are considered to be ‘worst-case scenarios’ with respect to difficulty of detection [40] in contrast with real-world interactions that may include univariate effects, and/or lower-order interactions that may facilitate the discovery of the overall interaction effect. In each simulated dataset, predictive and non-predictive features are known beforehand. Unlike the original benchmarking study that primarily used datasets with only 20 features, we increase the minimum feature count in this study to 100 for a more robust evaluation, except in exclusive-or (XOR) datasets. These XOR experiments model pure, strict, and clean 2-way to 5-way non-linearly separable interactions with full penetrance [41]. “Clean”, refers to data with no noise (i.e., heritability = 1; lower heritabilites are termed “noisy”). Thus, these XOR datasets serve as straightforward toy examples of feature interactions. Feature counts for these XOR datasets range from 20 to 100 in 20-feature increments. This setup results in 20 distinct experiments across four orders of interaction and five feature levels, enabling a systematic evaluation of each RBA’s ability to detect low and high-order interactions as feature counts increase. The XOR datasets contain the only clean interactions simulated in this project, yet all simulated interactions are both pure and strict. These datasets are available upon request, and are similar to datasets publicly available on GitHub [42] that were used in previous benchmarking [13].

**Table 1:** Simulation study datasets. 30 replicates of each configuration are generated. ‘Simulation method’ is either ‘C’ (custom script) or ‘G’ (GAMETES). ‘Configuration Variations’ describes further variations to a given dataset group. For example, 50:50/75:25 refers to the ratio of instance subgroup prevalence in heterogeneous problems

Simulated data group	Configurations	Config. variations	Predictive features	Total features	Model difficulty	Heritability	Instances	Simulation method
Description or pattern of association								
XOR Model	20	2-way,	2	20, 40, 60, 80, 100	N/A	1	1600	C
(Clean, *n*-way Epistasis)		3-way,	3					
		4-way,	4					
		5-way	5					
Core Datasets	32	-	2	100	E,	0.05,	200,	G
(Noisy 2-Way Epistasis)					H	0.1,	400,	
						0.2,	800,	
						0.4	1600	
Number of Features	3	-	2	100,	E	0.4	1600	G
(Noisy 2-Way Epistasis)				1,000,				
				10,000				
				100,000				
4-Feature Heterogeneous	2	50:50,	2	100	E	0.4	1600	G
2-Way Epistasis		75:25						
Noisy 3-Way Epistasis	1	-	3	100	E	0.2	1600	G
1-Feature Univariate Effect	8	-	1	100	E,	0.05,	1600	G
(Non-Epistatic)					H	0.1,		
						0.2,		
						0.4		
2-Feature Additive Effect	2	50:50,	2	100	E	0.4	1600	G
(Non-Epistatic)		75:25						
4-Feature Additive Effect	1	-	1	100	E	0.4	1600	G
(Non-Epistatic)								

### Experimental evaluation

We replicate the evaluation method from the previous benchmarking study [13] to compare ranking performance between methods. As negative controls, we add a random shuffle method that randomly shuffles the features to yield their rankings as well as a non-RBA (mutual information) best suited to detecting univariate effects. Specifically, we used a scikit-learn implementation of mutual information [43] with default settings. Thus, we explore a total of ten different methods: the standard and absolute value rankings of the four RBAs as well as random shuffle and mutual information (each with 30 replicate dataset analyses).

For each dataset configuration: (1) The predictive features are known ahead of time. For each of the 30 replicate datasets and ranking schemes, we find the ranking of the **lowest ranked** predictive feature. For example, in a dataset with 100 features and 2 predictive features, if the RBA ranks the predictive features as 1st and 5th, we save the 5th position. In total, we obtain 300 of such rankings (30 per method). Only the worst ranked positions, or the “weakest links”, are considered, as missing even a single predictive feature can cause RBAs to fail on feature selection problems with interactions. (2) Given *n* features in a dataset, there are *n* possible ranking positions. For each position, we compute the percentage of the 30 saved rankings that were placed **higher** than that particular position. This process is repeated for each method, and in total, we obtain 10*n percentages (*n* per method). (3) For analysis and visualization, we create heatmaps that summarize the experiments across all ten methods. Each heatmap consists of ten rows (one for each of the eight RBA methods (standard and absolute ranking), as well as random shuffle and mutual information). Each row has *n* grid squares. The percentages computed in the previous step are represented in the heatmap, where low percentages are encoded as orange-white and high percentages are blue.

In general, an effective feature scoring and ranking method would work towards maximizing the percentages calculated in this analysis. High percentages indicate that the saved predictive feature rankings are above most of the other feature positions. That is, all of the predictive features are consistently being placed in the top rankings, which is the desired result for RBAs. Therefore, a more intensely and consistently blue row in the heatmap signifies a higher performance level.

## Results

### Clean XOR low and high-order epistasis

**Fig. 1:**
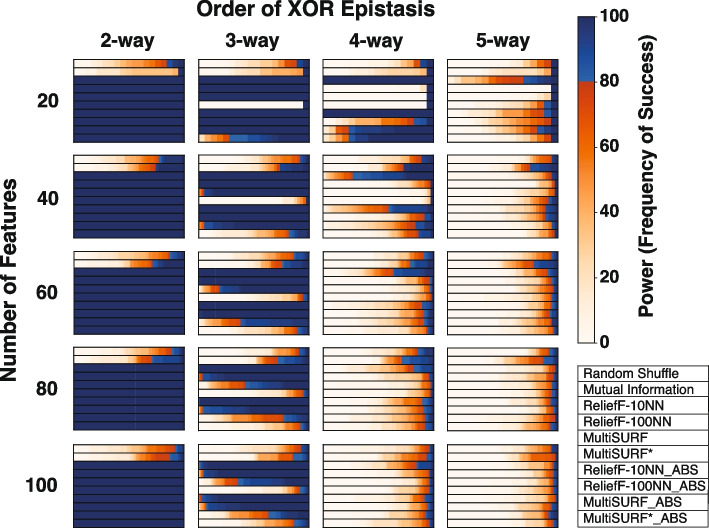
Heatmap results for pure and clean XOR epistatic datasets with increasing epistatic order and feature counts. The scale for power, as the frequency of success, is to the right of the heatmaps. Plot legend is located on the bottom right

Figure 1 shows results for the clean XOR datasets with increasing epistatic order and total feature count. All RBAs perform equally well at detecting predictive features for 2-way epistasis at all feature counts in contrast with Mutual Information and the random shuffle negative control. However, in 3-way datasets, only ReliefF-10NN and ReliefF-100NN, using both standard and absolute ranking, consistently show high power as feature count increases with Relief-10NN yielding the highest observed power. MultiSURF predicts well at 20 features, but performance starts to diminish as features are added with standard MultiSURF always outperforming MultiSURF_ABS. Interestingly, both MultiSURF rankings perform better at 100 features than at 80 features but only marginally so. MutliSURF* struggles in 3-way experiments but MultiSURF*_ABS outperforms standard MultiSURF* in all feature counts (only marginally so at 40 features and above). This occurs because standard MultiSURF* ranks predictive features with more negative scores.

In 4-way experiments at 20 features, standard ReliefF-10NN, ReliefF-10NN_ABS, MultiSURF_ABS, and MultiSURF*_ABS display high power. However, the power of these RBAs immediately diminishes in data with 40 features and continues to diminish as more features are added. By 100 features, the power of RBAs to detect predictive features in 4-way datasets is negligible, comparable to the power of the random shuffle. This drop-off is steeper for MultiSURF_ABS and MutliSURF*_ABS compared to ReliefF-10NN. Finally for 5-way datasets with 20 features, standard ReliefF-10NN, standard MultiSURF*, ReliefF-10NN_ABS, ReliefF-100NN_ABS, and MultiSURF_ABS display marginal power in contrast with the random shuffle with standard ReliefF-10NN achieving the highest power. However, as seen in 4-way, performance sharply diminishes as more features are added with only marginal power levels at 100 features. Unexpectedly, Mutual Information provides some marginal power in 4-way and 5-way experiments, especially when feature counts are intermediate (40-80 features). ReliefF-10NN_ABS consistently outperforms ReliefF-100N_ABS in higher-order interactions. This observation is surprising since Relief-100NN_ABS is expected to perform better in higher-order experiments as non-predictive features should be scored near zero, leaving opportunity for informative features to be scored more negatively. Indeed, we do observe average scores of non-predictive features in 4-way and 5-way XOR experiments closer to zero for ReliefF-100NN_ABS (0.0019) compared to ReliefF-10NN_ABS (0.0061). However, this trend also holds true for predictive features with ReliefF-100NN_ABS scoring predictive features closer to zero on average in 4-way and 5-way experiments (ReliefF-10NN_ABS: 0.011, ReliefF-100NN_ABS: 0.0024). Thus, unexpectedly, ReliefF-10NN_ABS showcases better performance in higher-order XOR interactions compared to ReliefF-100NN_ABS.

### Noisy 2-way epistasis

**Fig. 2:**
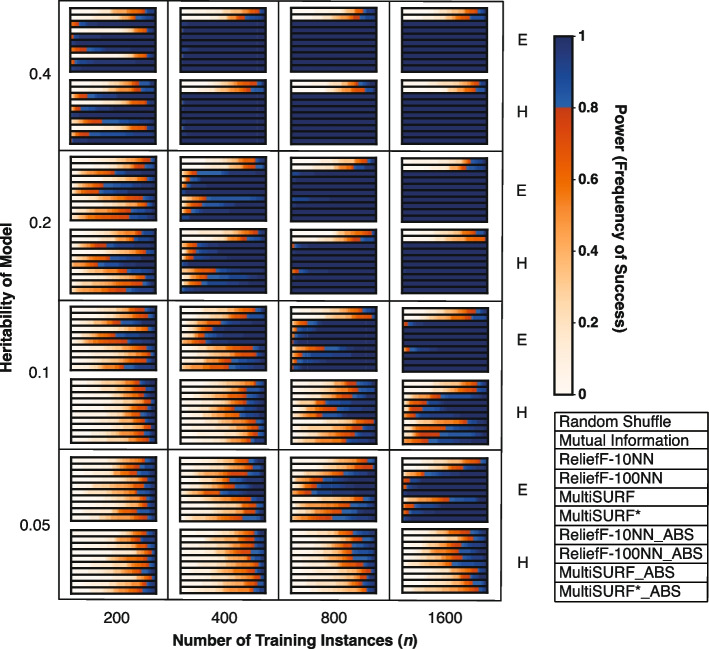
Heatmap results for noisy 2-way epistatic (core) datasets with varying levels of heritability and training instances. The scale for power, as the frequency of success, is to the right of the heatmap. Plot legend is located on the bottom right. E and H stand for easy and hard model architecture difficulty, respectively

Figure 2 shows results for the core set of 2-way epistatic interaction datasets with varying heritability (noise) and training instances. These datasets are comparable to the core 2-way datasets in the original benchmarking paper [13] but with a higher feature count (100 vs. 20) and the addition of absolute value rankings. Datasets that are the most difficult are towards the bottom left of the figure, with low heritability (high noise) and few training instances, while datasets which have high heritability (low noise) and more training instances are on the top right. The standard ranking performs slightly better in very noisy problems. Also, for very noisy problems, increasing the number of training instances increases the performance of the standard ranking substantially, but not as much for the absolute value methods. We find that RBAs assign predictive features with an absolute value very close to zero in noisier datasets. In contrast, both ranking methods generally assign much larger positive values to predictive features in less noisy problems. As a result, when there is less noise, using absolute ranking does not harm the ability to detect predictive features in these experiments. However, in very noisy datasets, where all feature scores are close to zero, with no very positive or very negative values, absolute ranking confounds ‘slightly good’ and ‘slightly bad’ features, making standard ranking perform better. Despite these trends, MultiSURF* is observed to have the highest power levels in most difficult dataset configurations when compared to other RBAs. Additionally, we observe that ReliefF-10NN outperforms ReliefF-100NN in dataset configurations with 200 training instances. However, ReliefF-100NN is superior as training instances increase.

**Fig. 3:**
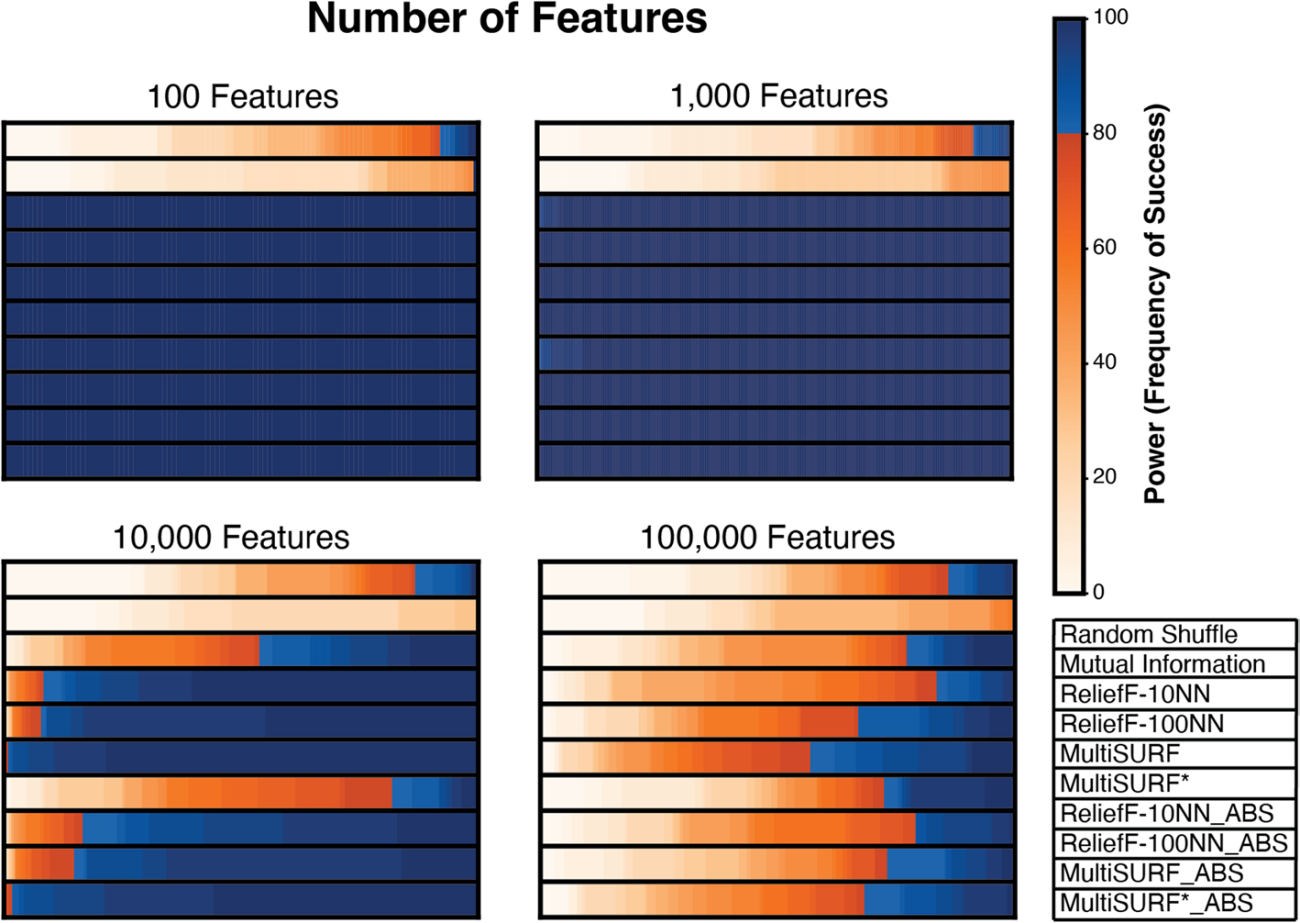
Heatmap results for detecting noisy 2-way epistatic interactions with a heritability of 0.4 and increasing non-predictive features. The scale for power, as the frequency of success, is to the right of the heatmap. Plot legend is located on the bottom right

Figure 3 shows results for 2-way epistatic datasets where total feature count increases by orders of magnitude from 100 to 100,000 and heritability set at 0.4. The standard and absolute ranking methods perform similarly well for datasets with 100 and 1000 features. At 10,000 features, standard ranking slightly outperforms absolute ranking in all RBAs with MultiSURF* having the highest power. However, MultiSURF*_ABS performs nearly as well as standard MultiSURF* at this feature count. This relationship is not observed at 100,000 features, where standard ranking outperforms absolute value ranking for MultiSURF and MultiSURF*. Standard MutliSURF* has the highest power at this feature count with MultiSURF*_ABS outperforming all other absolute value rankings as well. Thus, MultiSURF* may excel over other RBAs when feature counts are very high for 2-way epistasis detection. ReliefF-10NN struggles at 10,000 features. However, at 100,000 features ReliefF-10NN slightly outperforms ReliefF-100NN. Interestingly, absolute value rankings of both ReliefF-10NN and ReliefF-100NN outperform their respective standard rankings at 100,000 features due to these RBAs assigning more negative values to predictive features using the standard ranking. Thus, in very large datasets, using an absolute value ranking scheme may be optimal when employing ReliefF.

**Fig. 4:**
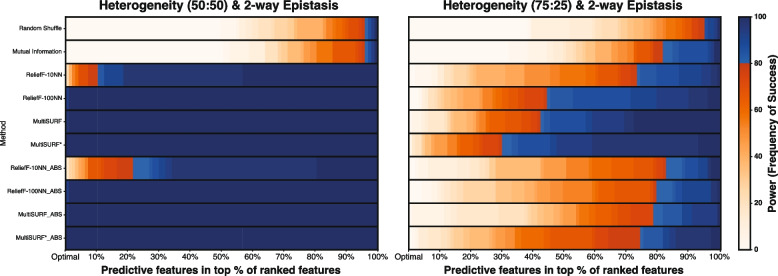
Heatmap results for detecting two independent heterogeneous 2-way epistatic interactions with a heritability of 0.4. The left heatmap shows results for a 50:50 split (equal subgroup prevalence), and the right heatmap shows results for a 75:25 split (unequal subgroup prevalence). The scale for power, as the frequency of success, is to the right of the heatmap

Figure 4 presents results for datasets that model heterogeneity between two independent 2-way interactions. This means there are two subgroups of instances, with one 2-way interaction being predictive for one subgroup of data instances, and the other 2-way interaction being predictive for the remaining data instances. One dataset models equal subgroup prevalence (50:50) while the other models unequal subgroup prevalence (75:25). Both the standard and absolute value rankings perform similarly well in the equal prevalence case. However, standard ranking significantly outperforms absolute value ranking in the unequal prevalence case. An exception to this is ReliefF-10NN as it has the lowest power in both subgroup prevalences with standard ranking outperforming absolute value ranking.

Upon further inspection of feature score outputs for the unequal prevalence dataset (75:25), we notice that the four RBAs with standard ranking give the two predictive features associated with the more prevalent subgroup highly positive scores, the two predictive features associated with the less prevalent subgroup scores closer to zero, and the non-predictive features slightly negative scores. This explains the poor performance of absolute ranking as non-predictive features will be close in value to features of the less prevalent subgroup when the absolute value is taken. The predictive features associated with the less prevalent subgroup received scores very close to zero because they are seen by the algorithm as non-predictive for most of the instances, which decreases their score. Taken together, predictive features associated with the less prevalent subgroup are ranked above the non-predictive features when using standard ranking, but below or similar using absolute value ranking. Notably, MultiSURF* achieves the highest power in the 75:25 experiment in both standard and absolute value ranking comparisons.

### Noisy 3-way epistasis

**Fig. 5:**
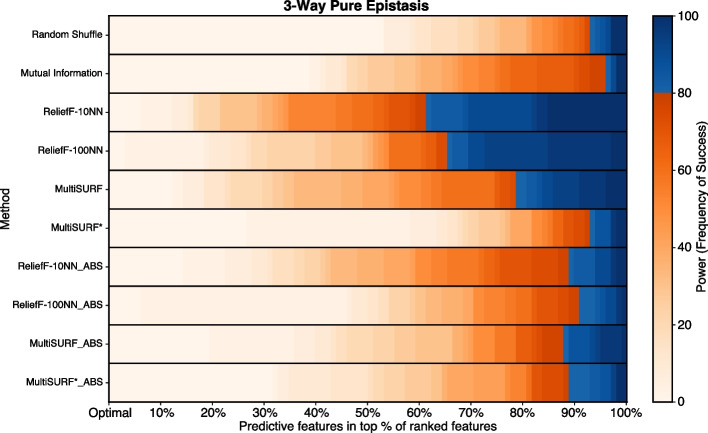
Heatmap results for noisy 3-way epistatic datasets with a heritability of 0.2. The scale for power, as the frequency of success, is to the right of the heatmap

All RBAs struggle to detect a 3-way epistatic interaction with a heritability of 0.2 (Fig. 5). Notably, a heritability of 0.2 is used here rather than 0.4 due to a known limitation of the GAMETES simulator [13]. Standard RBA rankings, except those of MultiSURF*, display intermediate power in this experiment. These intermediate results are likely due to the lower heritability of the model. MultiSURF*_ABS marginally outperforms standard MultiSURF*, which is also observed in the XOR experiments, for the same reason - predictive features are given negative scores by standard MultiSURF*.

### Non-epistatic associations

Standard and absolute rankings performed similarly for all non-epistatic experiments with the major exception being MultiSURF* (File S1). As previously observed, MultiSURF* struggles to detect single locus and additive effects as it is tailored to detect feature interactions [13, 22]. However, in non-epistatic experiments with more than one predictive feature (additive models), MultiSURF*_ABS outperforms MultiSURF* (File S1). This is due to MultiSURF* giving predictive features negative scores in some replicates, which increase in ranking when the absolute value is taken. Additionally, in the single feature (single univariate effect) experiment with varying levels of heritability and model architecture difficulty, ReliefF-100NN outperforms ReliefF-10NN at low heritability levels under the hard model architecture, however, the difference is marginal.

## Discussion

This study was inspired by results from our previous benchmarking study [13], where predictive features for higher-order epistasis received highly negative scores from most RBAs using the standard ranking approach. These results, and the work of Robnik-Šikonja and Kononenko [30], led us to hypothesize that absolute value ranking could effectively rank these features alongside main, additive, and lower-order epistatic effects. However the results of this study support the overall rejection of this hypothesis, more clearly defining the limitations of existing RBA algorithms with respect to higher order interactions (i.e., > 3-way epistasis).

In the original benchmarking study [13], only 20 features were used in most experiments, except for a noisy 2-way experiment that increased the feature count from 100 to 100,000. This study has replicated many of these simulations and experiments increasing the baseline feature count to 100 features in order to more robustly compare traditional feature ranking with absolute value ranking. An exception to this are the XOR experiments, which were designed to provide pure, strict, and clean toy examples of how increasing feature counts affect the detection of higher-order epistasis. These XOR experiments highlighted the (now apparent) limitations of RBAs in detecting higher-order interactions without the need to explore larger feature spaces. Our results show that RBAs using an absolute value ranking can only reliably detect higher-order interactions when penetrance and/or heritability is high (low noise) and feature count is low (Fig. 1). Thus, we only recommend employing absolute value ranking in these RBAs for these specific conditions. Interestingly, standard ReliefF-10NN displays the highest power levels and the least power decay across 4-way simulated XOR datasets as feature count increases. It also demonstrates the highest power in the simulated noisy 3-way epistasis experiment (Fig. 5). Therefore, when feature counts are low, ReliefF-10NN, using a standard ranking, could be a viable “best option” for 3-way and higher-order epistasis. However, even when these optimal conditions are met, all RBAs struggle to detect 5-way interactions, regardless of feature count, RBA algorithm, and the ranking method employed.

Typically, real-world studies exploring epistasis consider large sets of features, reflecting the extensive genomic datasets now available [8, 16]. Our experiment with increasing feature count, up to 100,000 features in datasets with 2-way interactions (Fig. 3), effectively replicated the previous observation of how feature space size limits RBA epistasis detection power [13]. Notably, wrapper algorithms such as TuRF [44] have been show to dramatically boost the performance in detecting 2-way interactions with individual RBA algorithms in larger feature spaces. However, we don’t expect TuRF (or similar RBA wrappers) to improve the detection of 4-way or higher interactions when combined with absolute value ranking, given that this ranking was only effective in data with very small feature counts.

Another replicated observation is the performance of MultiSURF* in 2-way epistasis experiments. When conditions are easy (low noise and lower feature counts), all RBAs perform similarly well. However, when conditions become more difficult, as noise and feature count increase, MultiSURF* provides the highest power in most configurations (Figs. 2, 3 and 4). Thus, as previously observed [13], it is recommended to employ MultiSURF* when investigating 2-way epistasis alone, especially in more challenging conditions.

In summary, the above results replicate many of the previous findings in RBA benchmarking [13], while definitively revealing the inherent limitations of these RBAs to detect higher order interactions (i.e., > 3-way) when applying either standard or absolute value ranking. While a niche “special case scenario” was identified where absolute value ranking improved performance to detect 4-way interactions it is unlikely this will be particularly useful in practice. A such, to date there are still no reliable and efficient methods for detecting higher order interactions in datasets with a large feature space, and that can be applied to data with different outcomes (e.g., binary, multi-class, continuous-values), handle missing data, and both categorical and quantitative features. This highlights the need to develop algorithms (including RBAs) that can flexibly conduct feature selection and/or detect high order interactions in datasets with a large feature space.

## Conclusions

In this study, we explore the effectiveness of multiple RBAs, with standard vs. absolute value ranking, in identifying predictive features involved in low vs. higher-order epistatic interactions (e.g., 2, 3, 4, and 5-way). Our findings clearly define RBAs overall inability to reliably detect 4 and 5-way interactions. Notable exceptions are the use of standard ranking with ReliefF-10NN and absolute value ranking with ReliefF-10NN, MultiSURF and MultiSURF*, which displayed intermediate to high power in fully penetrant 4-way XOR interactions, but only in datasets with 20 or so features. Therefore, these RBAs exhibit substantial limitations in detecting interactions beyond 3-way. Developing RBA approaches specifically tailored to detect higher-order interactions could be a promising direction for future research. Additionally, future research will focus on investigating whether these limitations persist across other models of epistasis other than XOR or GAMETES-simulated genetic datasets. We will also investigate comparisons between wrapper algorithms, including TuRF [44], IterRelief [45], and VLS Relief [46], to determine the strengths and weaknesses of these approaches with respect to detecting both low and high order interactions with standard and absolute ranking. Finally, we aim to examine if novel RBAs and/or alternative ranking schemes may have the ability to better identify higher-order interactions.

## Supplementary Information

Supplementary Material 1.

## Data Availability

Simulated datasets used in this work are available upon request. Similar datasets are publicly available on GitHub at https://github.com/EpistasisLab/rebate-benchmark. All analysis scripts are available in the v0.7.1 release of skrebate at https://github.com/UrbsLab/scikit-rebate.

## Abbreviations

ML: Machine Learning
NN: Nearest Neighbors
RBA: Relief-Based Algorithm
