# Supplementary material for: Assessing the limitations of relief-based algorithms in detecting higher-order interactions
Source: BioData Min. 2024 Oct 1;17:37. doi: 10.1186/s13040-024-00390-0 (PMC11443793; doi:10.1186/s13040-024-00390-0)
Supplement: Supplementary file 1 — Supplementary Material 1. [file 13040_2024_390_MOESM1_ESM.docx]

**Assessing the Limitations of Relief-Based Algorithms in Detecting Higher-Order Interactions**

**Freda, P.J., Ye, S., Zhang, R., Moore, J.H., and Urbanowicz, R.**

**Supplemental File 1 – Non-Epistatic Results**
